# Predicting global invasion risks: a management tool to prevent future introductions

**DOI:** 10.1038/srep26316

**Published:** 2016-05-20

**Authors:** D. H. Fletcher, P. K. Gillingham, J. R. Britton, S. Blanchet, R. E. Gozlan

**Affiliations:** 1 UMR MIVEGEC IRD-CNRS-Universités de Montpellier 1 et 2, Centre IRD de Montpellier, B.P. 64501, Montpellier, France; 2 Department of Life and Environmental Sciences, Faculty of Science and Technology, Bournemouth University, Poole, BH12 5BB, United Kingdom; 3 Centre National de la Recherche Scientifique (CNRS); Station d’Ecologie Théorique et Expérimentale, UMR5321, Moulis, 09200 Saint-Girons, France; 4 Centre National de la Recherche Scientifique (CNRS), Université Paul Sabatier, École Nationale de Formation Agronomique (ENFA); UMR5174 EDB (Laboratoire Évolution & Diversité Biologique), 118 route de Narbonne, F-31062 Toulouse cedex 4, France; 5 Unité Mixte de Recherche Biologie des Organismes et Écosystèmes Aquatiques (IRD 207, CNRS 7208, MNHN, UPMC) Muséum National d’Histoire Naturelle, 75231 Paris Cedex, France

## Abstract

Predicting regions at risk from introductions of non-native species and the subsequent invasions is a fundamental aspect of horizon scanning activities that enable the development of more effective preventative actions and planning of management measures. The Asian cyprinid fish topmouth gudgeon *Pseudorasbora parva* has proved highly invasive across Europe since its introduction in the 1960s. In addition to direct negative impacts on native fish populations, *P. parva* has potential for further damage through transmission of an emergent infectious disease, known to cause mortality in other species. To quantify its invasion risk, in regions where it has yet to be introduced, we trained 900 ecological niche models and constructed an Ensemble Model predicting suitability, then integrated a proxy for introduction likelihood. This revealed high potential for *P. parva* to invade regions well beyond its current invasive range. These included areas in all modelled continents, with several hotspots of climatic suitability and risk of introduction. We believe that these methods are easily adapted for a variety of other invasive species and that such risk maps could be used by policy-makers and managers in hotspots to formulate increased surveillance and early-warning systems that aim to prevent introductions and subsequent invasions.

The introduction and establishment of species into novel ecosystems can result in rapid dispersal rates^1^,^2^, with the magnitude of subsequent impacts and associated management costs increasing sharply with the time since the initial introduction^3^. When eradication is no longer a feasible management option, long-term strategies aimed at control and containment are generally adopted^4^,^5^. Such approaches tend to be expensive and only prevent further damage rather than restoring the invaded systems to their former status^6^. Consequently, more effective management strategies focus more on preventing introductions rather than managing subsequent invasions^7^.

Through increased global connectivity and trade, opportunities for the introduction of non-native species have increased^8^, with resources at border control stretched and ineffective at preventing the entry of non-native species into new regions^9^. Whilst risk assessment frameworks can be a strong mechanism to help allocate finite resources between preventing, detecting and managing introductions of non-native species^10^,^11^, they sometimes fail to highlight specific areas within regions where risks of introduction and subsequent invasions are higher^12^ (although, a notable exception is the recent application of the FISK risk assessment tool to a single catchment in Hungary^13^). Risk maps summarize landscape suitability for non-native species within a region by incorporating multiple factors, such as climatic or habitat suitability and potential introduction pathways, and thus can highlight smaller spatial areas within the region, or continent, where the risk is greatest^14^.

Freshwater ecosystems provide societies with valuable goods and services, yet are disproportionately vulnerable to biological invasions, compared to terrestrial systems, due to their high degree of isolation and endemism^15^,^16^. Introductions of non-native fishes represent a considerable threat to global freshwater fish conservation^17^ and can cause considerable economic damage^16^. For example, in North America the annual cost of invasive non-native fish species is estimated as at least US$ 5.4 billion^18^. In Europe, at least 650 fish species have been introduced into aquatic ecosystems, yet only a relatively small proportion of these are considered invasive and cause detrimental impacts^19^, such as the topmouth gudgeon *Pseudorasbora parva* (Temminck & Schlegel). This Asian cyprinid fish has been described as Europe’s most invasive freshwater fish (e.g.^20^), having achieved a pan-continental distribution in less than forty years, following its initial accidental introduction into Romania in the 1960s^21^. Its ability to invade new waters stems from traits that include high tolerance to degraded ecosystems, high reproductive effort and early sexual maturity alongside batch spawning and paternal nest guarding^22^. Moreover, it can rapidly colonize new waters^23^, with their larval stages particularly likely to disperse into the wider environment^24^. It is also a healthy carrier of an emergent infectious fungal disease *Sphaerothecum destruens* that has been implicated in declines in native European fishes^25^,^26^. Given its rapid rate of invasion in Europe and its disease risk, it is thus important to develop tools to predict additional locations at risk, outside of its current invasive range. Such tools would enable countries that contain potentially suitable habitats but have not yet been invaded to set up the necessary infrastructure to prevent an introduction and subsequent invasion.

Invasion risk is a combination of the likelihood of the species being introduced (e.g. the presence of an introduction pathway) and the suitability of the environment in the new region (e.g. climate suitability for the species). Consequently, the aim of this study was to predict the risk of *P. parva* invasion at the global scale in relation to its climate suitability and likely introduction pathways. Objectives were to (i) use climatic and other relevant abiotic data in both *P. parva*’s native and European invasive ranges to determine its realized niche; (ii) assess its potential global introduction pathways in relation to its known introduction pathways in Europe; and (iii) develop a niche model that characterizes its realized niche and combine this with data on introduction pathways to develop a global risk map of *P. parva* invasion. The implications of the risk map are then discussed in a global context.

## Materials and Methods

### Data on presence and absence of *P. parva*

Previous niche modelling studies suggest that presence location data from the invasive range can sometimes provide a significantly better approximation of the actual species niche breadth, thus producing more accurate suitability predictions beyond the native range^27^,^28^. Consequently, our presence dataset incorporated data from the European invasive range, compiled from European national monitoring schemes with consistent recording over the last 30+ years, as well as data from its native Asian range. When the European national monitoring data were combined with literature review and native and invasive presence records from the Global Biodiversity Information Facility (GBIF, www.gbif.org), this provided 2,882 confirmed *P. parva* observations from unique locations, across both native and invasive ranges, the latest of which was recorded in 2014. In order to minimise bias caused by multiple presence points in grid cells, i.e. spatial clustering of records, we applied a form of spatial filtering similar to that used by Boria *et al*.^29^, whereby we removed all but one record from grid cells, essentially leaving us with 2,048 “presence cells” in our dataset.

Absence data were generated on the principle of implied absence, as *P. parva*’s European distribution, after 40 years since introduction, suggested the species had already reached saturation point (*cf*.^21^) and we also assumed equilibrium in the native range. To avoid spatial autocorrelation with presence cells, random points were generated outside buffers of 50 km around presence cell centroids. These random points were limited to a maximum distance of 100 km from presence cell centroids to prevent the characterisation of areas far outside the species’ core areas, which can result in poor model performance^30^. These methods also maintained a degree of parity with regard to the ratio of presence to absence points across the two distributional aggregations (Asian and European). A total of 10,000 absence points were generated in 10 absence datasets, and weighted to give equal prevalence for the overall response variable dataset, which was coded in a binary (1/0, presence/absence) fashion. Due to the relatively high recording effort achieved in this monitoring, it was assumed these locations represented genuine absences, rather than just characterising the background environmental variation present within the invasive range.

### Predictor variable data

We used a suite of abiotic variables as a basis for the description of *P. parva*’s niche. We used the 19 Bioclimatic datasets (Bio1 - Bio19^31^, see Table 1 for individual descriptions); Mean Potential Incoming Solar Radiation (INMSR); Topsoil pH (TpH); and Topographic Wetness Index (TWIS) as our initial suite of variables (see Table 1 for details of datasets). So as to promote parsimony and minimize over-fitting in models, we refined the list of variables used in the final modelling procedure, using Variance Inflation Factor to identify and remove collinearity in the final suite of variables used. Stepwise selection of variables was carried out using the r package ‘VIF’^32^ and left us with a refined suite of ten abiotic variables (see variables marked with asterisk in Table 1) to use in our models. All datasets were used at a spatial resolution of 10 km and a spatial reference of GCS WGS 1984. Conforming to accepted protocols in ecological niche modelling^33^, all of the remaining ten variables are ecologically relevant to *P. parva*.

### Additional data

In order to produce a risk map accurately representing both the likelihood of introduction and climatic suitability, we incorporated a dataset representing the known major introduction pathways. Given that the spread of *P. parva* throughout Europe was strongly related to aquaculture-related introductions^21^, and because there is a strong correlation between non-native introductions and international trade^34^, the “Aquaculture Pressure” dataset from the “Global Threats to Human Water Security and River Biodiversity” database^35^ was used as a surrogate for introduction likelihood. This dataset was derived using national annual average aquaculture harvests from inland and diadromous fishes from 1997–2006 from the UN Food and Agriculture Organization’s FishStat Plus database (www.fao.org/fishery), which were then distributed proportionately to the grid-cell specific discharge for each nation. The original dataset was re-sampled using bilinear interpolation to match the resolution of all other datasets (10 km). All spatial datasets shared the same spatial reference (GCS WGS 1984).

### Modelling methods

The first phase of modelling used nine modelling techniques: Artificial Neural Networks (ANN), Classification Tree Analysis (CTA), Flexible Discriminant Analysis (FDA^36^), Generalized Additive Models (GAM), Generalized Boosted Models (GBM), Generalized Linear Models (GLM), Multivariate Adaptive Regression Splines (MARS), Random Forests (RF) and Surface Range Envelope (SRE^37^). The use of GLM, GAM, CTA and ANN is described and discussed in Thuiller^38^. GBM incorporates interactive relationships between predictor variables, as well as being able to discern complex response curves^39^. MARS and ANN are good at deciphering complex relationships^40^ and RF models have been found to perform consistently well when evaluated against various other well established methods^41^. The models were implemented in the ‘R’ software^42^, using the ‘biomod2’ R package^43^. Modelling parameters were kept at default values, in the interest of repeatability. Model predictions were output as probability of presence (continuous values between 0 and 1).

Evaluation of model accuracy (predictive power) was carried out using the Area Under Curve (AUC) of the Receiver Operating Characteristic (ROC). A random 70% selection of the data was used to train each model before the remaining 30% of data were used to evaluate accuracy. To compensate for any possible spatial autocorrelation between training and evaluation data^44^, this cross-validation process was repeated ten times for each model, with the mean calculated to provide a value for a version of the model trained using all of the data.

The final ensemble forecast output was produced in ‘biomod2’ using a weighted average technique, based on the AUC values of each model. Models with higher AUC values were more influential in deciding final cell values. Evaluation of final ensemble model accuracy was conducted using the same cross-validation approach as that used for individual models. Variable importance for each individual model was assessed through the use of biomod2’s randomization function, where each variable’s values are randomized in turn. Pearson’s correlations of model predictions before and after randomization were used to infer relative importance of the variable being randomized^44^. Where the correlation is high, the relative importance of the variable to the prediction outcome is low. Final Ensemble Model (EMmw) variable importance was calculated by applying the weightings used for the ensemble forecasting to the variable importance scores for individual models, summing values by variable then dividing by the number of models used.

### Creation of the *P. parva* risk map

The final risk map was created by multiplying the values of the EMmw layer, which represents suitability of conditions according to modelled realized niche, by those of the “Aquaculture pressure” layer, which represents likelihood of introduction. Both layers’ values range from 0–1, so the final Risk Map represents equal weightings of the two factors in assessment of the risk of successful invasion, with potential values ranging from 0 (no risk) to 1 (high risk).

## Results

### Predictive accuracy in models

**Figure 1:**
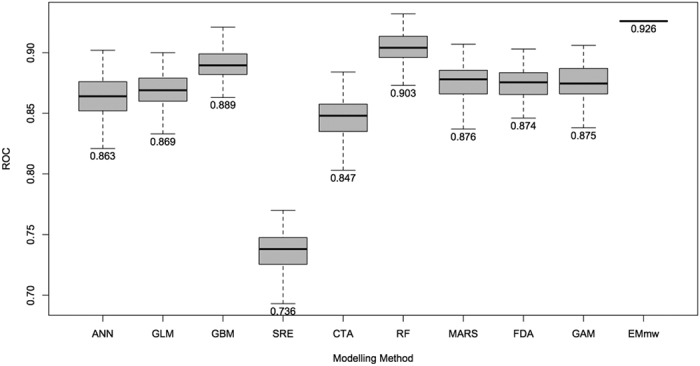
Box-plots displaying the Area Under Curve (AUC), Receiver Operating Characteristic (ROC) evaluation scores for all models, grouped by modelling method. Components of box-plots represent minimum, lower quartile, mean upper quartile and maximum values for each modelling method. For each group n = 100, except for EMmw, where n = 1.

Predictive accuracy across the nine individual modelling methods was consistently high and the mean of each modelling method always exceeded 0.847 in cross-validation runs, except for SRE (Fig. 1). SRE models were dropped from the final EM, as they were consistently lower scoring than the others. AUC scores indicated that all of the retained individual models were acceptable^45^ for predicting the climatic suitability of locations for *P. parva*. However, the weighted method of ensemble forecasting provided a significant increase in model accuracy (Welch’s *t*-test *p *≤ 0.001, testing results of all retained individual models against ensemble model). Variability of predictive accuracy across the different absence datasets and the randomized subsets of data (i.e. partitioning for training and testing) was very similar across all modelling methods, indicating that the data for training were representative of the total dataset in each instance (i.e. for each absence dataset and for each randomization run, of which there were 100 in total for each modelling method; 900 in total).

### Predictor variable importance

**Figure 2:**
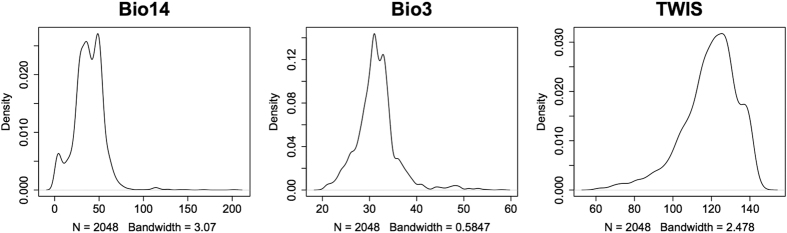
Density plots of top three most important variables in final mean-weighted Ensemble Model (EMmw), in order of importance; (**a)** Bio14 – Precipitation of driest month (mm); (**b)** Bio3 – Isothermality (mean diurnal temperature range divided by annual temperature range; (**c)** TWIS – Topographic Wetness Index.

The three most important predictor variables, Bio14, Bio3 and TWIS (in that order) accounted for 66% of the sum of all importance values (Table 2). The density plot curve for Bio14, precipitation of driest month, (Fig. 2) suggested that *P. parva* prefer areas with values between 25 and 60 mm. The density plot for Bio3, isothermality, suggested that *P. parva* prefers areas with a mean monthly temperature range that is approximately 33% of the total annual range. Topographic wetness index was the most important of the non-‘bioclim’ variables used in the final modelling, ranking 3rd with a mean score of 11.64% in the randomization tests.

### Suitability predictions

**Figure 3:**
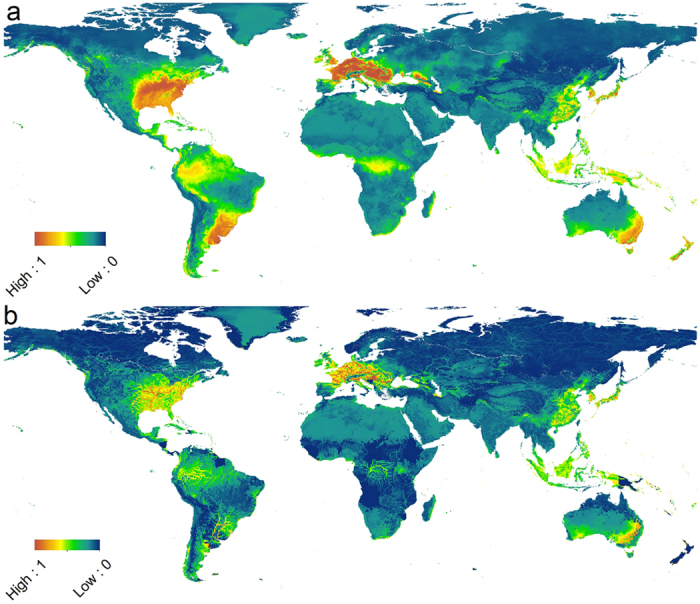
(**a,b**) Suitability (**a**) and Risk (**b**) maps. Both maps use the same colour scale, from low value of 0 to high value of 1, indication niche suitability and risk of successful invasion, respectively. Final layout of map panels was generated using Esri ArcMap (Version 10.0, Build 2414. url: https://www.arcgis.com/).

Current known ranges, both invasive (Europe) and native (Asia), were generally well represented by the model output (Fig. 3a). However there were some areas within the native range where the suitability score was lower than expected, such as Taiwan. In addition to the known current invasive range, a number of other areas displayed medium to high suitability values. These areas included parts of central and southern Africa, Malaysia, Indonesia, New Guinea, New Zealand, Australia, North and South America. The highest values outside the current known range were in the USA (predominantly in the western and southern states), South America (focused around southern Brazil, eastern Paraguay and Argentina, and all of Uruguay), in Eastern Australia and in New Zealand.

### Risk predictions

The process of incorporating introduction likelihood to the map refined the suitability map by producing a prediction of relative likelihood of a successful invasion (Fig. 3b). Areas with predicted high suitability and high levels of aquaculture pressure, a surrogate for introduction likelihood, were fairly widespread. The most significant areas of high and medium-high risk values included the USA, Brazil, Paraguay, Uruguay, Argentina, coastal South Africa, Malaysia, Indonesia and Australia. Although New Zealand possessed areas with high suitability values, the low values of aquaculture pressure meant that the risk levels were very low throughout the country. Similarly, the Island of New Guinea possessed medium-high suitability values, but aquaculture values in the country of Papua New Guinea meant that the eastern part of the island received generally low risk values, whilst the western part of the island, a region of Indonesia with higher levels of aquaculture, received a medium-high risk of successful invasion.

## Discussion

The risk maps indicated that *P. parva* has a substantial scope to invade areas and regions beyond its current invasive range, and these cover all continents. There were several hotspots for climatic suitability and risk of introduction in each continent that could represent a potential future point of entrance. In North America for example, the key states at risk are Mississippi, Louisiana, Arkansas, N/S Carolina, Georgia, Alabama, and Tennessee. In South America, two geographically separated hotspots were identified, which included medium-high values in upper parts of the Amazon basin in Peru and Columbia and Brazil. Further south, high values were observed in the south of Brazil, Uruguay and north-east parts of Argentina. In Africa there were narrow fringes of medium-high risk in coastal areas of South Africa, Madagascar and in Liberia. However these were surrounded by more diffuse areas of medium risk, generally following the coast but also present in central countries such as the Democratic Republic of the Congo and Uganda, where the upper part of the Congo River is most at risk. Although already present in East Asia, our outputs indicated that areas in northern India would be suitable for *P. parva*, particularly the foot hills of the Himalayas, including the Indus River basin as far west as Srinagar in the Jammu and Kashmir state of India. Of particular note is the recent discovery of *P. parva* in the Brahmaputra River in India^46^, an area predicted to be climatically suitable and at medium-high risk by our analysis, thus providing confidence in the performance of the risk maps, particularly given that our training dataset contained only one presence cell in the entirety of India. Finally, in Oceania, predicted areas at medium-high to high risk were in Indonesia, Malaysia and parts of eastern Australia.

Evaluation of the model via cross-validation techniques indicated that the modelling method used to predict climatic suitability achieved a high degree of accuracy. However, there were areas where high suitability values were expected, due to known presence of *P. parva*, but were not predicted by the model. For instance there were locations in Belarus and southern Turkey that received low suitability scores, but contained *P. parva* records – although they are only present in relatively low abundances^47^,^48^. Equally, in the native range, Taiwan received generally low suitability scores, despite being known to host widespread *P. parva* populations^21^. This disparity between predicted and observed suitability in Taiwan may have been due to underrepresentation of distinct environmental conditions present there, owing to low numbers of presence points in this location. Such an issue can lead to underrepresentation of these conditions in the modelled niche. Because niche conservatism is a fundamental assumption of the ecological modelling techniques, the projected niche may not have included the full range of suitable conditions in Taiwan.

In time, introduced species will adapt and evolve within their new range, potentially shifting their climatic preferences and their ecological niche. Simon *et al*.^49^,^50^ revealed *P. parva* populations of Japan and Taiwan have been isolated from those found in continental China for some time (5–6 MYA and 1–1.5 MYA, respectively), allowing the adaptation of the Taiwanese populations to the more tropical climate there. Neither the Japanese, nor the Taiwanese populations have been introduced to the European invasive range, which could explain why the climatic niche of the invasive range may have not yet included the distinct part of the niche occupied only by the Taiwanese populations, which would compound the underrepresentation of this part of the niche in the data used to train our models. One implication of this is that the risk of invasion may be heavily dependent upon the source population for the introduction, particularly when the native ecological niche is distinct compared to that of other populations. In the case of the Taiwanese populations, this may mean that scope for successful invasion into areas with more tropical climates is greater, and for the species as a whole, this may mean that current risk predictions are underestimated for tropical regions.

Despite some of the invasion predictions in India already being realized, the risk level of individual countries will also be determined by extant policies on managing non-native species regarding aspects such as risk assessments and surveillance^4^. For example, the high level of awareness in Australia regarding non-native species introductions^51^ and the calls for more consistent use of appropriate risk assessment tools, as long ago as the 1990s (e.g.^52^), should have by now resulted in adoption of such practices for all major groups of taxa. However, particularly with regard to non-native fishes, this has not been the case; moreover the only application of a generic risk assessment scheme for non-native fishes, to date, has been that of Vilizzi and Copp^53^, in 2013. With this in mind, the addition of species-specific risk maps, such as the one produced in our study, present an important additional tool that can identify the specific regions of a country that are most at risk from an introduction and thus enable increased preventative measures such as import bans and additional screening at entry points to be implemented^54^,^55^. This increased prevention is preferable, as it should reduce the long-term management costs^3^. Currently, screening points for non-native species are often at ports or national/state borders^7^, locations which might not necessarily be identified as having a high risk of introduction and invasion but are entry points for species in transit and so can serve as useful points of inspection.

Failing to prevent future invasions of *P. parva* would greatly increase the associated threat to fish biodiversity. This species is a healthy carrier of a severe emerging fungal pathogen, *S. destruens*^24^, that is non species-specific (i.e. generalist) and has so far led to disease in all tested species including salmonids, cyprinids and percids^25^,^56^. Because of the life history traits that make *P. parva* invasive in so many locations (e.g. broad tolerances, high fecundity, etc) and because it is a healthy carrier of the disease (i.e. it doesn’t suffer mortality from infection), it constitutes a significant threat, as it can rapidly build dense populations which can act as persistent disease reservoirs for an outbreak. A recent study has highlighted the particular significance of the threat posed by such species^57^. Given the long distance of natural salmon migrations, the potential impact of an outbreak of *S. destruens* among North American salmonid populations (which are highly sensitive to the disease^58^,^56^), could have far reaching consequences. *P. parva’s* invasion could thus be the potential stepping stone to a much wider transmission of the disease. We now know that the rate of spread after initial introduction in Europe was about 0.5 countries per year^21^, and that invasion in a region comprising largely suitable areas is likely to result in an array of negative impacts.

Given the potential impacts of *P. parva* of disease transmission and the formation of highly abundant populations that can lead to increased inter-specific competition^59^, there are strong ecological drivers for the avoidance of introduction. Whilst simple climate-matching approaches can be useful, more representative models such as ours, accounting for critical factors such as introduction likelihood/ propagule pressure (*cf*.^60^,^23^) should be used in order to provide more accurate risk representations. Consequently, the outputs in this paper can be used to formulate stronger preventative measures and ensure the invasion of *P. parva* is limited to Europe and does not develop more globally.

## Additional Information

**How to cite this article**: Fletcher, D. H. *et al*. Predicting global invasion risks: a management tool to prevent future introductions. *Sci. Rep*. **6**, 26316; doi: 10.1038/srep26316 (2016).

## Figures and Tables

**Table 1:** Description of abiotic descriptor datasets used in analysis along with source URLs.

GIS data layer	Short name	Source URL
Annual Mean Temperature	Bio1*	http://www.worldclim.org/bioclim
Mean Diurnal Range (Mean of monthly (max temp - min temp))	Bio2*	http://www.worldclim.org/bioclim
Isothermality (BIO2/BIO7) (*100)	Bio3*	http://www.worldclim.org/bioclim
Temperature Seasonality (standard deviation *100)	Bio4	http://www.worldclim.org/bioclim
Max Temperature of Warmest Month	Bio5	http://www.worldclim.org/bioclim
Min Temperature of Coldest Month	Bio6	http://www.worldclim.org/bioclim
Temperature Annual Range (BIO5-BIO6)	Bio7	http://www.worldclim.org/bioclim
Mean Temperature of Wettest Quarter	Bio8 *	http://www.worldclim.org/bioclim
Mean Temperature of Driest Quarter	Bio9	http://www.worldclim.org/bioclim
Mean Temperature of Warmest Quarter	Bio10	http://www.worldclim.org/bioclim
Mean Temperature of Coldest Quarter	Bio11	http://www.worldclim.org/bioclim
Annual Precipitation	Bio12	http://www.worldclim.org/bioclim
Precipitation of Wettest Month	Bio13	http://www.worldclim.org/bioclim
Precipitation of Driest Month	Bio14*	http://www.worldclim.org/bioclim
Precipitation Seasonality (Coefficient of Variation)	Bio15	http://www.worldclim.org/bioclim
Precipitation of Wettest Quarter	Bio16	http://www.worldclim.org/bioclim
Precipitation of Driest Quarter	Bio17	http://www.worldclim.org/bioclim
Precipitation of Warmest Quarter	Bio18 *	http://www.worldclim.org/bioclim
Precipitation of Coldest Quarter	Bio19 *	http://www.worldclim.org/bioclim
Mean potential incoming solar radiation (8-day average) derived in SAGA GIS	INMSR*	http://www.worldgrids.org
Topsoil pH (H2O) based on the Harmonized Worlds Soil Database	TpH*	http://www.worldgrids.org
SAGA GIS Topographic wetness index	TWIS*	http://www.worldgrids.org

Variables in the column entitled short name marked with an asterisk were retained for modelling procedures.

**Table 2:** Mean variable importance by modelling method, as a percentage.

Model	Bio1	Bio2	Bio3	Bio8	Bio14	Bio18	Bio19	TpH	INMSR	TWIS
ANN	12.50	4.33	8.96	6.79	30.11	12.43	16.46	0.42	2.54	5.44
GLM	3.05	2.89	22.36	6.63	41.50	1.17	5.00	0.79	4.37	12.24
GBM	4.36	2.42	25.89	1.65	46.73	1.63	1.08	0.03	2.05	14.16
SRE	10.08	10.12	10.11	10.45	7.28	9.60	9.37	10.52	11.25	11.22
CTA	9.87	5.64	20.10	4.67	34.33	5.24	3.60	0.60	3.52	12.43
RF	12.62	5.62	24.48	3.98	18.69	6.21	6.15	2.51	7.86	11.88
MARS	7.50	5.48	17.18	5.25	41.93	1.26	6.96	0.10	3.10	11.24
FDA	7.48	3.50	22.83	5.42	35.04	1.42	4.70	0.38	6.70	12.54
GAM	7.85	4.01	30.64	6.74	15.49	3.75	11.60	1.27	5.61	13.05
EMmw	8.15	4.23	**21.60**	5.13	**32.94**	4.13	6.93	0.77	4.48	**11.64**

Variable importance scores, as measured by randomization technique, calculated for individual models as 1— Pearson’s correlation between predictions, before and after randomization. Scores were then converted into a % of the sum of all variable importance scores for each modelling method. The three most important variables in the final EMmw model are highlighted in bold.
